# Recent advances in producing food additive L‐malate: Chassis, substrate, pathway, fermentation regulation and application

**DOI:** 10.1111/1751-7915.14206

**Published:** 2023-01-05

**Authors:** Qiang Ding, Chao Ye

**Affiliations:** ^1^ School of Life Sciences Anhui University Hefei China; ^2^ Key Laboratory of Human Microenvironment and Precision Medicine of Anhui Higher Education Institutes Anhui University Hefei China; ^3^ Anhui Key Laboratory of Modern Biomanufacturing Hefei China; ^4^ School of Food Science and Pharmaceutical Engineering Nanjing Normal University Nanjing China

## Abstract

In addition to being an important intermediate in the TCA cycle, L‐malate is also widely used in the chemical and beverage industries. Due to the resulting high demand, numerous studies investigated chemical methods to synthesize L‐malate from petrochemical resources, but such approaches are hampered by complex downstream processing and environmental pollution. Accordingly, there is an urgent need to develop microbial methods for environmentally‐friendly and economical L‐malate biosynthesis. The rapid progress and understanding of DNA manipulation, cell physiology, and cell metabolism can improve industrial L‐malate biosynthesis by applying intelligent biochemical strategies and advanced synthetic biology tools. In this paper, we mainly focused on biotechnological approaches for enhancing L‐malate synthesis, encompassing the microbial chassis, substrate utilization, synthesis pathway, fermentation regulation, and industrial application. This review emphasizes the application of novel metabolic engineering strategies and synthetic biology tools combined with a deep understanding of microbial physiology to improve industrial L‐malate biosynthesis in the future.

## Graphical abstract

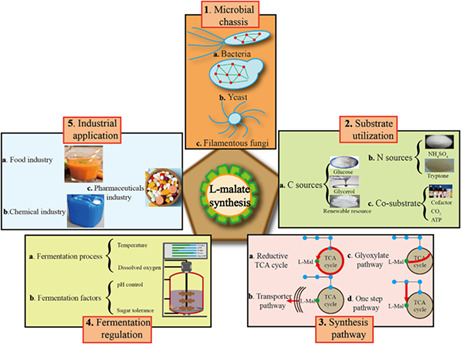

## INTRODUCTION

L‐malate, also known as 2‐hydroxy succinate, is a widely used food additive, as well as a valuable chemical and pharmaceutical intermediate (Chi et al., 2016; Iyyappan, Baskar, et al., 2019; Iyyappan, Bharathiraja, et al., 2019; Wang et al., 2013). As one of the C4‐dicarboxylic acids, L‐malate has a number of high‐value applications (Cao et al., 2020; Werpy, 2004). However, problems related to environmental pollution and resource waste are limiting the further application of chemical synthesis of L‐malate (Ahn et al., 2016; Ferone et al., 2019; Luo et al., 2021). Thus, new strategies for L‐malate synthesis should be economical and environmentally friendly (Li, Shen, et al., 2021; Li, Yang, et al., 2021).

**FIGURE 1:**
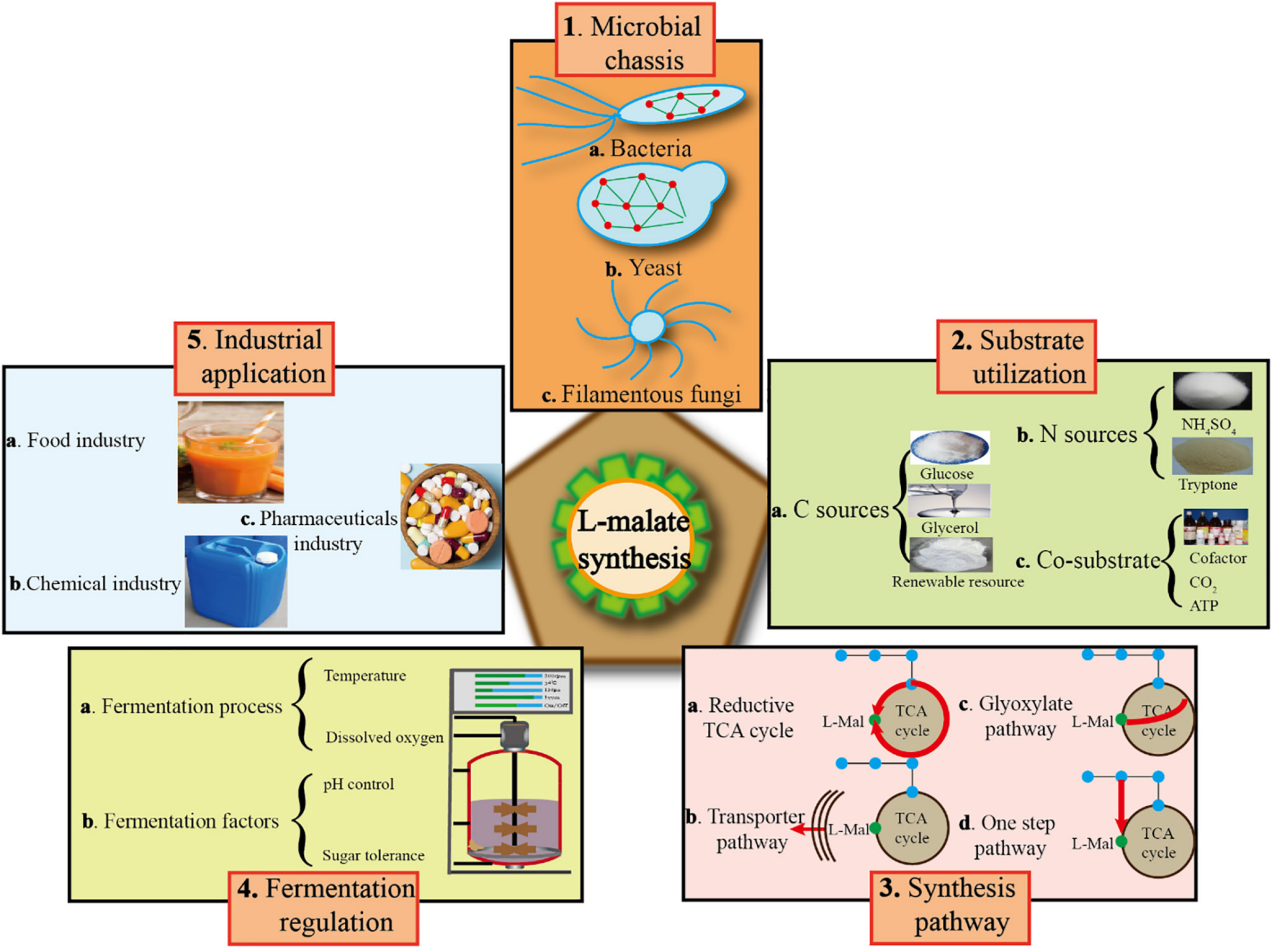
Recent advances in producing L‐malate chassis, substrate, and pathway. The main advances were involved in microbial chassis, substrate utilization, synthesis pathway, fermentation regulation and industrial application.

In this review, we comprehensively summarized recent advances in biotechnological approaches for L‐malate biosynthesis, including the microbial chassis (e.g. bacteria, yeast, *Aspergillus* sp., and other chassis strains), substrate utilization (e.g. carbon sources, nitrogen sources, and other substrates), synthesis pathway (TCA cycle, transport, and other pathways), fermentation regulation (process engineering and other fermentation factors), as well as industrial application (food additive, chemical, pharmaceutical industry). Furthermore, we discuss the future prospects of improving L‐malate synthesis by constructing a more efficient CO_2_ sequestration system, developing *Aspergillus oryzae* as a novel chassis, improving the tolerance of the strain to environmental stress conditions, compartmentalizing key enzymes to enhance the biotransformation, and decoupling cell growth from L‐malate synthesis (Figure 1).

## MICROBIAL CHASSIS STRAINS FOR L‐MALATE BIOSYNTHESIS

The selection of an optimal microbial chassis is crucial for the production of target chemicals with high titre, yield, and productivity (Liu et al., 2020; Luan et al., 2020; Matsumoto et al., 2017; Ye & Chen, 2021). Thus, the pipeline for the construction of microbial chassis strains for L‐malate production needs to be outlined in detail (Calero & Nikel, 2019; Vickers et al., 2010). Accordingly, promising chassis strains based on bacteria, yeast, and *Aspergillus* sp. that can efficiently synthesize L‐malate through metabolic engineering or biochemical strategies will be discussed (Table 1).

**TABLE 1:** Microbial chassis used for L‐malate production.

Category	Strains	Regulation strategies	Yield (g/g)	Titre (g/L)	Productivity (g/L/h)	References
Bacteria chassis	*B. subtilis* 168	The *ldhA* and *pta* were deleted, *mdh* and *pepc* were expressed	–	2.10	0.03	Mu & Wen 2013
	*E. coli* XL‐1	The *scfA* and *pykF* were expressed	–	12.08	0.14	Somasundaram et al. 2018
	*E. coli* XZ658	The *ldhA, ackA, adhE, pflB, mgsA, poxB,frdBC, sfcA, maeB, fumB* and *fumAC* were *deleted, mdh* and *pck* were expressed.	1.06	34.00	0.47	Zhang et al., 2011
	*E. coli* GL2306	The *adhE, ackA, ldhA, pts1,pflB, focA* and *mgsA* were deleted, *pck* and *mdh* were expressed	0.39	25.86	0.36	Guo et al. 2018
	*E. coli* KJ071	The *ldhA, adhE, ackA, focA, pfB* and *mgsA* were deleted, *ppc* and *mdh* were expressed	1.04	69.10	0.48	Jantama et al. 2008
Yeast chassis	*T. glabrata* T.G‐PMS	The *mae* and *mdh* were expressed	–	8.50	0.14	Chen et al. 2013
	*Schizophyllum commune* IFO‐4928	Wild type, fermentation optimization	–	21.00	0.19	Mikio et al. 1997
	*C. glabrata* 012	The *pyc* and *mdh* were expressed, CgUSV1and CgYAP3 were interacted with CgMed16.	–	35.50	0.33	Liang et al. 2021
	*S. cerevisiae* W4209	The *pyc*, *mae* and *mdh* were expressed	0.31	30.75	0.32	Chen et al. 2017
	*Pichia pastoris* Pp‐PC‐MDH1	The *pyc* and *mdh* were expressed	–	42.28	0.45	Zhang et al. 2015
	*S. cerevisiae* RWB525	The SLK was deleted, The *pyc* and *mdh* were expressed	0.31	59.00	0.19	Zelle et al. 2008)
	*A. pullulans* strain ZX‐10	Acidolysis of PMA	0.55	144.20	0.74	Zou et al. 2013
	*U. trichophora* TZ1	Evolutionarily strain, fermentation optimization.	–	196.00	0.74	Zambanini, Sarikaya, et al. 2016
	*A. oryzae* DSM1863	Wild type, fermentation optimization	–	58.2	0.16	Ochsenreither et al. 2014
Filamentous fungi	*P. sclerotiorum* K302	Wild type, fermentation optimization	0.88	92.00 (calcium malate)	1.23	Wang et al. 2013)
	*A. flavus*	Wild type, fermentation optimization	0.94	113	0.59	Battat et al. 1990
	*R. delemar* HF‐121	Wild type, fermentation optimization	–	120.00	2.00	Li et al. 2014
	*P. viticola* 152	Wild type, fermentation optimization	1.28	168.00 (calcium malate)	1.75	Khan et al. 2014
	*A. niger* S1149	The *oahA* and *cexA* were deleted, *pyc*, *mdh3, c4t318, mstC, hxkA, pfkA, pkiA* were expressed	1.22	201.10	1.05	Xu, Liu, et al. 2020; Xu, Zhou, et al. 2020

### Bacterial chassis strains for L‐malate production

Bacteria used for synthesizing L‐malate mainly include *Escherichia coli* and different *Bacillus species*. *E. coli* is a mature host for heterologous gene expression and has been paid much attention by genetic engineering experts because of its clear genetic background, ease of manipulation, simple culture conditions, and economic large‐scale fermentation (Table 1) (Ding et al., 2021; Yang et al., 2021). However, wild‐type *E. coli* only produces L‐malate as a minor by‐product of central metabolism under the aerobic conditions (Ding et al., 2018). Thus, the fast growth and mature genetic tools of *E. coli* were utilized to design an L‐malate synthesis pathway through metabolic engineering, synthetic biology, and systems biology strategies (Calero & Nikel, 2019; Vickers et al., 2010). A typical strategy for L‐malate production in *E. coli* is to delete the byproduct synthesis genes and overexpress pathway enzyme genes. For example, *E. coli* KJ060 was derived from *E. coli* ATCC 8739 by deleting the *ldhA*, *ackA*, *adhE*, and *pflB* genes to avoid byproduct formation. Furthermore, the engineered strain XZ658 was constructed by knocking out *fumABC*, *maeB*, *sfcA*, *frdBC*, *mgsA*, and *poxB*, as well as overexpressing metabolic pathway genes in *E. coli* KJ060. Notably, the L‐malate titre, yield and productivity of *E. coli* XZ658 reached 21.84 g/L, 0.74 g/g and 0.30 g/L/h, respectively, and were further increased to 34 g/L, 1.06 g/g, and 0.47 g/L/h by applying a dual‐stage fermentation strategy with aerobic cell growth and anaerobic L‐malate production (Zhang et al., 2011).

As a GRAS (generally recognized as safe) organism, *B. subtilis* has already been used to construct cell factories for the production of food additives, enzymes, and health care products (Liu, Li, Du, et al., 2017; Liu, Li, Shin, et al., 2017; Liu, Xie, Shin, et al., 2017). In addition, many advanced system biology tools were developed in *B. subtilis*, including dynamic adaptation to environmental changes, sRNA and antisense RNA, enzyme–enzyme interactions, post‐translational regulation, and allosteric interactions (Liu, Li, Du, et al., 2017; Liu, Li, Shin, et al., 2017; Liu, Xie, Shin, et al., 2017). For example, a heterologous phosphoenolpyruvate carboxylase and L‐malate dehydrogenase were introduced into *B. subtilis* and lactate dehydrogenase was deleted to synthesize 1.27 g/L L‐malate (Mu & Wen, 2013). Finally, the L‐malate titre, yield and productivity were increased to 2.09 g/L, 0.12 g/g and 0.02 g/L/h through cell growth and product synthesis‐based two stages fermentation, representing improvements of 86.3%, 60% and 86.3%, respectively. Similarly, *Bacillus coagulans* is also considered a favourable microbial chassis for the biosynthesis of target products. To produce L‐malate in *B. coagulans*, the pyruvate carboxylase (*pyc*), L‐malate dehydrogenase (*mdh*), and phosphoenolpyruvate carboxykinase (*pckA*) genes were introduced to construct a reductive TCA cycle, while lactate dehydrogenase (*ldh1/ldh2*), acetoin synthase (*als*), and pyruvate formate lyase (*pflB*) were deleted to block competing pathways. Finally, the L‐malate titre, yield and productivity respectively reached 25.5 g/L, 0.3 g/g and 0.34 g/L/h in dual‐phase fed‐batch fermentation (Sun et al., 2021).

### Yeast chassis development for L‐malate production

Yeasts are well‐characterized and easily manipulated eukaryotic microorganisms that have been widely used for the industrial production of high‐value chemicals, such as organic acids, ethanol, fatty acids, and natural products (Table 1) (De et al., 2021; Wang et al., 2021). Recent advances in yeast cell factory development were mainly included harnessing natural subcellular organelles, developing genetic circuits, genome modification, adaptive laboratory evolution, protein degradation, and stress tolerance, which enable the construction of robust cell factories (Aranda‐Diaz et al., 2017; Jarque et al., 2016; Patra et al., 2021; Yu et al., 2018). In a large‐scale bio‐refinery, yeast can efficiently synthesize L‐malate to meet ever‐increasing demands. For example, the pyruvate carboxylase (*pyc*) and L‐malate dehydrogenase (*mdh*) were overexpressed to construct a reductive TCA cycle, which increased the L‐malate titre to 4.73 g/L. Next, the C4‐dicarboxylate transporter and *Spmae* deubiquitination were regulated to increase the L‐malate titre in *Saccharomyces cerevisiae* to 22.14 g/L. Furthermore, the gene expression level was further regulated by different strength promoters to obtain a titre‐yield and productivity of 30.75 g/L, 0.31 g/g and 0.32 g/L/h, respectively (Chen et al., 2017). In addition to *Saccharomyces cerevisiae*, non model yeasts such as *Torulopsis glabrata, Schizophyllum commune*, and *Zygosaccharomyces rouxii* also showed potential as efficient chassis strains for L‐malate production, titers reaching 8.5, 21, and 75 g/L, respectively (Chen et al., 2013; Mikio et al., 1997; Taing & Taing, 2006). In *Ustilago trichophora* yeast, the synthesis capacity can be improved through adaptive laboratory evolution to increase the growth rate and production rate by 2.5‐fold and 6.6‐fold, respectively. Furthermore, the titre, yield, and productivity of L‐malate can reach 196 g/L, 0.82 g/g, and 0.39 g/L/h in *Ustilago trichophora* TZ1 (Zambanini, Sarikaya, et al., 2016; Zambanini, Kleineberg, et al., 2016). Based on this, a metabolic engineering strategy was introduced in *U. trichophora*. For example, the *mdh1, mdh2, ssu1*, and *ssu2* genes were overexpressed to improve the L‐malate titre, yield and productivity from glycerol to 120 g/L, 0.54 g/g and 0.31 g/L/h, respectively, which was finally improved to 134 g/L, 0.42 g/g and 0.56 g/L/h in the bioreactor (Zambanini et al., 2017). This highly engineered strain of *U. trichophora* has been patented (Lars et al., 2018).

In addition, *Aureobasidium pullulans* is a potential non‐model strain for the production of L‐malate and poly(β‐L‐malic acid) (PMA). It is notable due to its very large genome encoding many hydrolases capable of degrading various plant materials as renewable substrates (Cao et al., 2012; Cao et al., 2013; Liu, Li, Du, et al., 2017; Liu, Li, Shin, et al., 2017; Liu, Xie, Shin, et al., 2017; Zou et al., 2013). Thus, L‐malate can be efficiently synthesized through metabolic engineering of *A. Pullulans* (Cheng et al., 2017; Zou et al., 2019; Zou et al., 2021). In addition to PMA hydrolysis, *A. pullulans* strain ZX‐10 was screened based on its light‐yellowish and creamy colonies in the PDA medium. Then, a fibrous‐bed bioreactor (FBB) was used to culture *A. pullulans* strain ZX‐10, resulting in a PMA titre, yield and productivity of 144.2 g/L, 0.55 g/g and 0.74 g/L/h in fed‐batch mode, respectively. Furthermore, PMA hydrolysis was achieved by sulfuric acid to produce free malate with a recovery rate of 84% (121.1 g/L) (Zou et al., 2013). In addition, soy molasses was utilized by *A. pullulans* strain ZX‐10 as a renewable substrate in conjunction with corn steep liquor (CSL) in a 5‐L stirred‐tank fermenter. Furthermore, NH_4_NO_3_ nitrogen limitation was optimized, resulting in an economically competitive L‐malate production process with a titre, yield and productivity of 71.9 g/L, 0.69 g/g and 0.29 g/L/h, respectively (Cheng et al., 2017; Wei et al., 2017).

### Filamentous fungi as chassis strains for L‐malate production

Although studies on bacteria and yeasts have achieved notable results, there is still no economically viable L‐malate production process due to its low yield. Filamentous fungi have a number of intrinsic advantages for L‐malate production, such as high acid tolerance, high protein titers, and native acid production (Li et al., 2020; Xu, Liu, et al., 2020; Xu, Zhou, et al., 2020). In addition, synthetic biological tools, the CRISPR/Cas9, gene assembly, dynamic regulation and pathway construction were successfully applied to transform the genus *Aspergillus* into an industrial chassis (Kim et al., 2016; Ko et al., 2020; Liu et al., 2020).

*Aspergillus flavus, A. niger, and A. oryzae* are three key species used to synthesize L‐malate. These strains possess a strong ability of protein decomposition and saccharification at the same time (Table 1) (Chi et al., 2016; Kovilein et al., 2021; West, 2011). In *A. flavus*, an L‐malate titre yield and productivity of 113 g/L, 0.94 g/g and 0.59 g/L/h were achieved by optimizing the medium and fermentation conditions. However, *A. flavus* can potentially produce the highly dangerous aflatoxin during the fermentation process, which complicates further downstream separation and raises concerns regarding food safety (Battat et al., 1990). As an industrial citric acid‐producing strain, *A. niger* can also produce L‐malate. For example, *A. niger* ATCC 10577 can use thin stillage as the substrate to obtain a 19 g/L titre of L‐malate, with a yield of 0.8 g/g and productivity of 0.01 g/L/h (West, 2011). In contrast to the abovementioned aspergilli, *A. oryzae* is a GRAS organism that can be used to produce food‐grade enzymes and organic acids. For example, the *mdh, pyc, pck*, and *ppc* genes were overexpressed to reinforce the key enzymes and glyoxylate cycle for constructing strain WS‐M‐P‐PP, which produced an L‐malate titre of 58.5 g/L titre with a productivity of 0.65 g/L/h, representing 38.3% improvements compared to strain WS‐M‐P expressing the *mdh* and *pyc* genes (Liu, Li, Du, et al., 2017; Liu, Li, Shin, et al., 2017; Liu, Xie, Shin, et al., 2017). In addition, the synthetic biological tools and metabolic engineering strategies, such as CRISPR techniques, DNA assembly, gene deletion, and so on, have already been developed and applied in different strains of *Aspergillus* to produce target chemicals. In addition to aspergilli, industrially relevant filamentous fungi also include *Penicillium sclerotiorum* K302, *P. viola*, and *Rhizopus delemar*, which can produce L‐malate titers of 92, 168, and 120 g/L, respectively (Khan et al., 2014; Li et al., 2014; Wang et al., 2013). These studies demonstrated that non‐model strains can also efficiently produce L‐malate with high titers through fermentation optimization and metabolic engineering. However, the cultivation and scale‐up of a filamentous fungus is much more difficult than yeast, which is mainly attributed to (i) The morphology of filamentous fungi, as the length of their mycelia can reach several hundred micrometres. Furthermore, pellet and mycelium morphology, process control and productivity are highly interlinked (Driouch et al., 2012; Veiter et al., 2018). Therefore, agitation in fermenters can easily affect the mycelial morphology of filamentous fungi, whereas yeast fermentation is rarely affected by morphology (Timoumi et al., 2018; Zhang et al., 2020); (ii) The spores of filamentous fungi can easily degenerate, resulting in the decline of viability and fermentation performance (Veiter et al., 2018); (iii) The conventional fermentation time of filamentous fungi is more than 5 days, even more than 10 days. This long fermentation time increases the risk of bacterial contamination and the cost of scale‐up (Liu, Li, Du, et al., 2017; Liu, Li, Shin, et al., 2017; Liu, Xie, Shin, et al., 2017).

## SUBSTRATE UTILIZATION FOR L‐MALATE BIOSYNTHESIS

Substrate utilization directly affects the fermentation cost, downstream separation, and microbial metabolism (Xu et al., 2009; Zhu & Tang, 2017; Zou et al., 2013). Accordingly, carefully selected carbon sources, nitrogen sources, and co‐substrates can be used to increase L‐malate biosynthesis.

### Carbon sources for L‐malate production

Conventional carbon sources, non‐conventional carbon sources, and intermediates lead to large differences in the performance of L‐malate fermentation (Ye et al., 2013).

Among conventional carbon sources, glucose and glycerol can be used for efficient L‐malate bio‐manufacturing. Glucose is the most widely distributed monosaccharide in nature, and it can be used by most microorganisms. Accordingly, glucose can also be used as the substrate for L‐malate biosynthesis. In a reported metabolic engineering strategy for the direct conversion of glucose into L‐malate, the malic enzyme and the non‐ATP‐forming Embden‐Meyerhof pathway were used to balance the redox cofactors during L‐malate synthesis. Finally, the engineered strain achieved an L‐malate titre yield and productivity of 0.35 g/L, 0.72 g/g, and 0.01 g/L/h, respectively, while the ATP turnover number reached 9, corresponding to a production rate of 0.02 mol/ml/min (Ye et al., 2013). In addition to glucose, crude and pure glycerol are efficient carbon sources for L‐malate production. Crude glycerol derived from biodiesel production is an economical substrate for the production of high‐value chemicals. A recent study used response surface methodology and artificial neural networks to find optimal conditions for L‐malate production from glycerol in *A. niger*, which resulted in an optimized growth rate of 0.1542 h^−1^, L‐malate titre of 92.64 g/L, and productivity of 0.48 g/L/h (Iyyappan, Baskar, et al., 2019; Iyyappan, Bharathiraja, et al., 2019). Some strains can also use pure glycerol to synthesize L‐malate, such as *Ustilago trichophora* TZ1. After adaptive laboratory evolution and medium optimization, this strain achieved an L‐malate titre yield and productivity of 195 g/L, 0.45 g/g, and 0.74 g/L/h, which was finally improved to 1.94 g/L/h under the optimal conditions (Zambanini, Sarikaya, et al., 2016; Zambanini, Kleineberg, et al., 2016).

The non‐conventional carbon source mainly include renewable raw materials, acetate, and xylose (Kovilein et al., 2021; Li, Feng, et al., 2018; Xia et al., 2017). Renewable raw materials mainly include abundant agricultural products or wastes, such as Jerusalem artichoke tuber hydrolysate and corn straw hydrolysate. As mentioned above, polymalic acid (PMA) is a water‐soluble polyester that is widely used in medicine and food. PMA production from Jerusalem artichoke tuber hydrolyzate was increased by upregulating pyruvate carboxylase and L‐malate dehydrogenase. As a result, the titre and yield of PMA reached 117.5 g/L and 0.7 g/L/h, while that of pullulan reached 15.2 g/L and 0.09 g/L/h using Jerusalem artichoke tuber as substrate in a 1000‐L fermenter, respectively (Xia et al., 2017). For L‐malate production from corn straw hydrolysate, *R. delemar* HF‐119 was used as the starting strain to screen high‐producing mutants, and the best strain achieved an L‐malate titre of 60 g/L. Based on further metabolic flux was analysis, the acid‐limited mutant *R. delemar* HF‐121 was developed from the alcohol‐limited mutant strain *R. delemar* HF‐120. Finally, the L‐malate titre of *R. delemar* HF‐121 reached 120 g/L within 60 h, corresponding to a productivity of 2.0 g/L/h, with a yield of 0.96 g/g (Li et al., 2014). Acetate is an abundant and inexpensive substrate, which can be used to regulate cell growth and produce target chemicals (Oswald et al., 2016). For example, the optimal acetate concentration of 45 g/L induced a favourable pellet‐like morphology in *A. oryzae* DSM 1863, resulting in an L‐malate titre, yield and productivity of 8.77 g/L, 0.19 g/g and 0.05 g/L/h from acetate as substrate, respectively (Kovilein et al., 2021). Xylose is the second most abundant sugar component of lignocellulosic biomass, which makes it one of the most abundant renewable resources in the world. To enable xylose utilization in strain MA‐13, D‐tagatose 3‐epimerase, L‐fructokinase, L‐fuculose‐phosphate aldolase, and aldehyde dehydrogenase A were overexpressed. After the byproduct and L‐malate synthesis pathways were optimized through metabolic engineering, the titre, yield and productivity of L‐malate reached 5.9 g/L, 0.8 g/g and 0.03 g/L/h, respectively (Li, Feng, et al., 2018). A comprehensive list of substrates used for L‐malate production in published studies can be found in Table 2.

**TABLE 2:** L‐malate production from various substrates by different microorganisms.

Category	Substrates	Strains	Yield (g/g)	Titre (g/L)	Productivity (g/L/h)	References
Conventional substrate	Glucose	*E. coli*	0.34 g/g (glucose)	9.25	0.77	Moon et al. 2008
	Glucose	*S. cerevisiae*	0.96 g/g (glucose)	11.8	0.38	Pines et al. 1997
	Glucose	*A. niger*	1.22 g/g (glucose)	201.24	1.05	Xu, Fei, et al. 2019; Xu, Shan, et al. 2019
	Glycerol	*U. trichophora* TZ1	0.26 g/g (glycerol)	118	0.75	Zambanini, Sarikaya, et al. 2016; Zambanini, Kleineberg, et al. 2016
	Glycerol	*A. niger*	0.18 g/g (glycerol)	83.2	0.43	Iyyappan et al. 2018
Non‐conventional substrate	Corn straw hydrolysate	*R. Delmar* HF‐119	0.96 g/g (corn straw hydrolysate)	120	2.0	Li et al. 2014
	Acetate	*A. oryzae* DSM 1863	0.19 g/g (acetate)	8.77	0.15	Kovilein et al. 2021
	Xylose	*E. coli* MA‐11	0.80 g/g (xylose)	5.90	0.08	Li, Feng, et al. 2018
	Thin stillage	*A. niger*	0.80 g/g (thin stillage)	19.00	0.09	West, 2011
	Beech wood cellulose hydrolysate	*A. oryzae* DSM 1863	0.97 g/g (Beech wood cellulose hydrolysate)	37.9	0.23	Dorsam et al. 2017
	Syngas	*A. oryzae* DSM 1863	0.17 g/g (syngas)	1.1	0.01	Oswald et al. 2016
	Corn starch	*A. oryzae*	0.90 g/g (corn starch)	117.20	1.17	Liu et al. 2018
	Cellulose	*Thermobifida fusca* muC‐16	0.63 g/g (cellulose)	62.76	0.51	Deng et al. 2016)

### Nitrogen sources for L‐malate production

Various organic and inorganic nitrogen sources can have a distinct influence on cellular metabolism and fermentation performance (Ji et al., 2021). Organic nitrogen sources mainly include tryptone and yeast extract. In a study using yeast extract, pyruvate carboxylase, L‐malate dehydrogenase and a malic acid transporter were overexpressed in *A. oryzae* 2103a‐68, resulting in a yield, volumetric productivity and maximum specific production rate of 1.11 g/g, 1.05 g/L/h and 0.25 g/g/DCW, respectively (Knuf et al., 2014). In a study using tryptone, a novel nitrogen source regulation strategy was established to separate a growth stage, acid‐producing stage, and autolysis stage for optimal L‐malate production. As a consequence, the titre, yield and productivity of L‐malate reached 164.9 g/L, 0.77 g/g and 1.14 g/L/h in a 30‐L fermenter, representing increases of 26.7%, 24.2%, and 27.8%, respectively (Ji et al., 2021).

In addition to organic nitrogen sources, inorganic (NH_4_)_2_SO_4_ can also be used to synthesize L‐malate, and effectively replace the expensive tryptone. In a study using (NH_4_)_2_SO_4_ as the sole nitrogen source to screen mutants obtained through atmospheric room temperature plasma (ARTP), ^60^Co‐γ irradiation, and diethyl sulfate (DES) mutagenesis, the mutant *A. oryzae* FMME218‐37 achieved a final L‐malate titre, yield and productivity of 95.2 g/L, 0.54 g/g and 0.57 g/L/h, respectively (Ding et al., 2018). Researchers also investigated the effect of nitrogen starvation conditions based on (NH_4_)_2_SO_4_ and tryptone as nitrogen sources, resulting in L‐malate titers of 22.27 and 30.27 g/L, respectively. Finally, the yeast transcription factor Msn2/4 and pyruvate carboxylase reaction were identified by transcriptome analysis for further metabolic engineering applications (Knuf et al., 2013). Furthermore, the carbon‐to‐nitrogen ratio also has a significant influence on the biosynthesis of industrial chemicals. When *A. oryzae* DSM 1863 was cultured using xylose and glycerol as the carbon sources and (NH_4_)_2_SO_4_ as the nitrogen source with a C/N ratio of 200:1, the nitrogen was consumed quickly in the early stage of organic acid production. As a result, the titre, yield and productivity of L‐malate respectively, reached 58.2 g/L, 0.52 g/g and 0.16 g/L/h, while 4.2 g/L fumarate were obtained at the same time (Ochsenreither et al., 2014).

### Co‐substrates for L‐malate production

**FIGURE 2:**
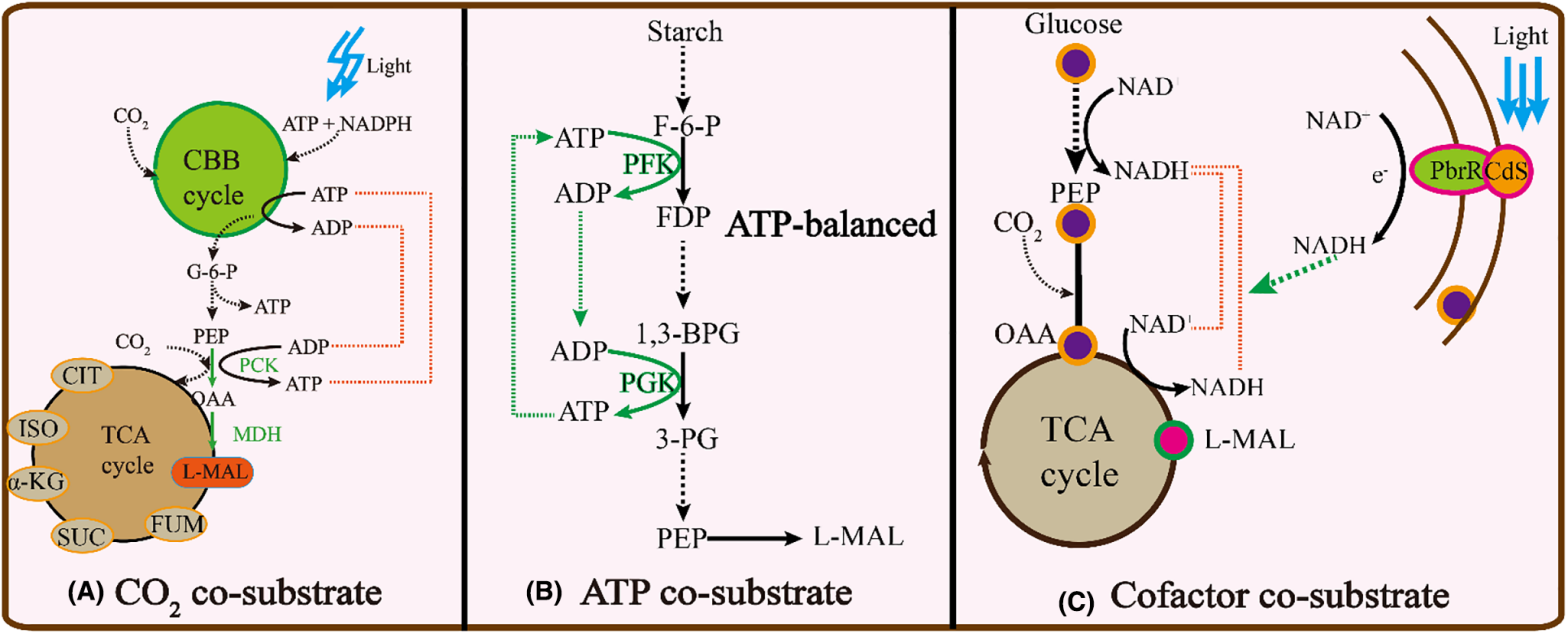
Co‐substrate for L‐malate production. The main advances were involved in CO_2_ co‐substrate, ATP co‐substrate, and cofactor co‐substrate for L‐malate production.

Co‐substrates of L‐malate synthesis mainly include CO_2_, ATP, and other cofactors (Hu et al., 2018). CO_2_ can assist the carbon flux toward target chemicals, which is mainly achieved by adding carbonate, introducing a heterologous CO_2_ fixation cycle, and engineering novel CO_2_ assimilation pathways. In addition to being a source of CO_2_, CaCO_3_ also acts as a buffer, which can reduce the negative effects of acid production. For example, an L‐malate titre of 57.71 g/L and productivity of 0.4 g/L/h were obtained by using single‐factor experiments and response surface methodology to optimize the CaCO_3_ concentration (Liu et al., 2014). In a different approach, the Calvin‐Benson‐Bassham cycle was introduced to increase CO_2_ utilization efficiency and carbon yield for L‐malate production based on glucose as the main carbon source (Figure 2). As a result, the CO_2_‐fixation rates of *E. coli* and *Synechococcus elongatus* were increased by 870% and 110%. Moreover, the L‐malate titre, yield and productivity were respectively increased to 51.86 g/L, 1.09 g/g, and 0.72 g/L/h in *E. coli*, as well as 0.19 g/L and 0.02 g/L/d in *Synechococcus elongatus* through an ATP‐generating and ATP‐consuming RuBisCO shunt (Hu et al., 2018). For engineering a novel CO_2_ fixation pathway, formate dehydrogenase, formyltetrahydrofolate cyclohydrolase, methylene tetrahydrofolate dehydrogenase, methylene tetrahydrofolate reductase, formaldehyde lyase (FLS), and dihydroxyacetone kinase were expressed in *E. coli*. When combined with a CdS‐nanodot light harvesting system, strain LF1 achieved an L‐malate titre, yield and productivity of 18.89 g/L, 1.22 g/g and 0.2 g/L/h (Hu et al., 2021).

The supply of ATP can be enhanced in vivo and in vitro to enhance L‐malate synthesis based on starch as carbon source (Shi et al., 2019). To increase the ATP supply in vivo, the *fumB, frdABCD, ldhA* and *pflB* genes were deleted, while *mdh* and *pck* were overexpressed in *E. coli* BA040 to construct *E. coli* BA063. This strain achieved an L‐malate titre of 28.5 g/L with a yield of 0.69 g/g and productivity of 0.43 g/L/h, while the intracellular ATP concentration reached 715.79 nmol/g DCW. In a study focusing on the production of L‐malate in vitro, a synthetic biosystem for the conversion starch and CO_2_ into L‐malate was implemented by designing an ATP‐balanced pathway. Using this system, an L‐malate titre, yield and productivity of 7.02 g/L, 0.15 g/g and 1.42 g/g were obtained in vitro (Shi et al., 2019).

In addition to ATP, the L‐malate synthesis pathway also requires NADPH and NADH (Dong et al., 2017). To increase the NADPH supply for malic enzyme as the key node of the pathway, the *pos5* gene encoding an NADH kinase was introduced to transform NADH into NADPH. After additionally blocking byproduct pathways through gene knockouts and introducing an NADP^+^‐dependent malic enzyme mutant, the L‐malate titre yield and productivity of the resulting strain *E. coli* F0931 respectively 21.65 g/L, 0.36 g/g and 0.04 g/L/h in a 5‐L fermenter (Dong et al., 2017). For increasing the NADH supply, a rhodopsin‐based light‐driven ATP regeneration system and a dynamic regulatory switch based on the cyanobacterial transcription factor NdhR were developed to reinforce the NADH pool and carbon yield for L‐malate synthesis. Finally, an L‐malate yield of 1.04 g/g was obtained by combining glucose as the main substrate with the CO_2_ sequestration system (Hu et al., 2021).

## SYNTHESIS PATHWAY OF L‐MALATE

**FIGURE 3:**
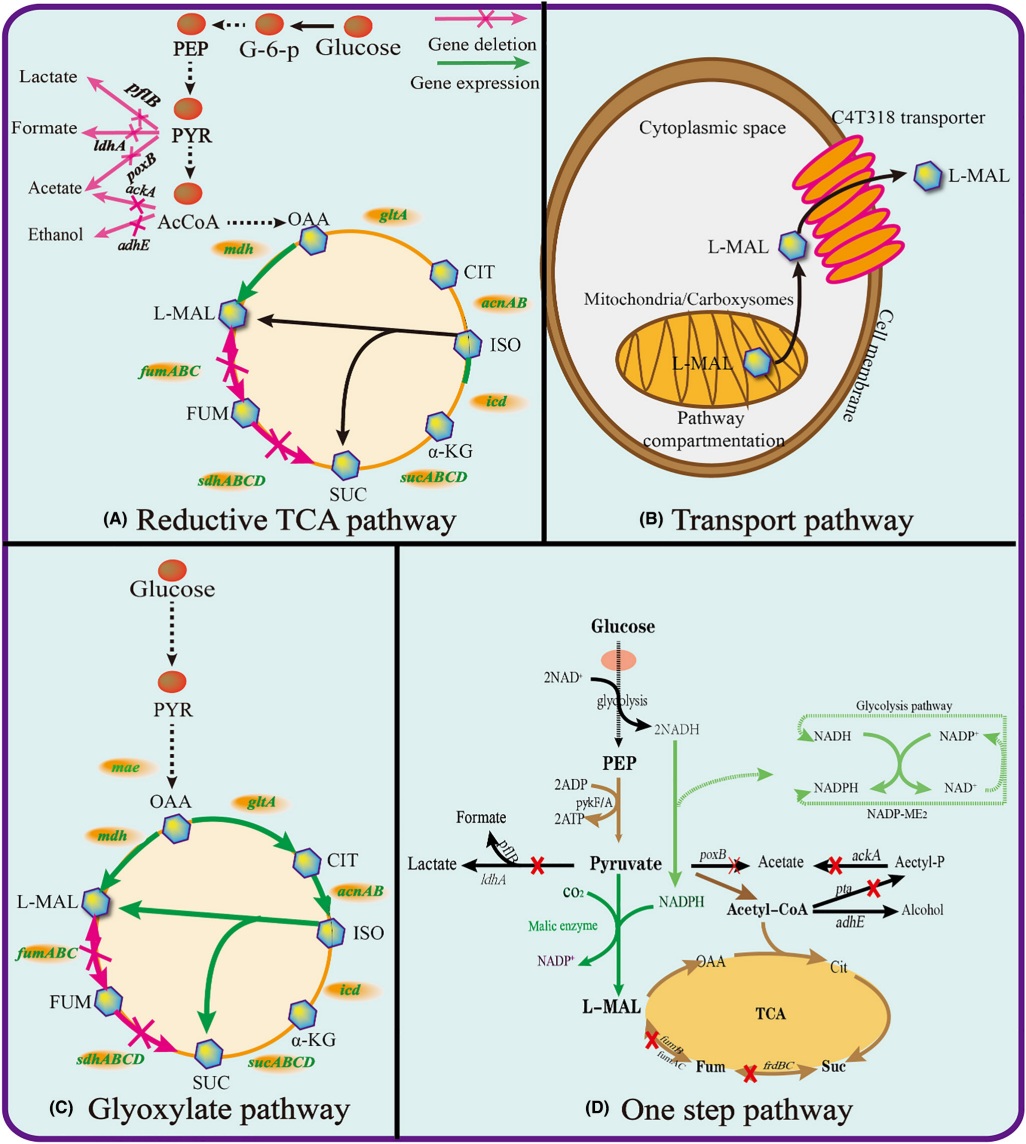
The metabolic pathway for L‐malate production. 1,3‐BPG; 3‐PG, 1,3‐diphosphoglycerate, 3‐phosphoglycerate; *aceA*, isocitrate lyase; *aceB*, malate synthase; *ackA*, acetate kinase gene; *acnAB*, aconitate hydratase; *adhE*, alcohol dehydrogenase gene; CIT, citrate; FUM, fumarate; *fumB*, fumarase; *gltA*, citrate synthase; ISO, isocitric acid; *ldhA*, lactate dehydrogenase gene; MAL, L‐malate; mdh, malate dehydrogenase; OAA, oxaloacetic acid; *pflB*, fFormic acid lyase gene; *poxB*, pyruvate oxidase gene; SCO, succinyl‐coA; *sdhABCD*, succinate dehydrogenase; SUC, succinate; *sucABCD*, succinyl‐CoA synthetase; α‐KG, α‐Ketoglutaric acid.

L‐malate is an intermediate of the TCA cycle, and its flux is affected by the Embden‐Meyerhof‐Parnas (EMP) pathway (Li, Shen, et al., 2021; Li, Yang, et al., 2021; Yin et al., 2015). Accordingly, the reductive TCA pathway, transporter engineering, glyoxylate pathway and one step synthesis pathway can be used for L‐malate biosynthesis (Figure 3).

### Reductive TCA pathway for L‐malate synthesis

Different microorganisms utilize either an oxidative or a reductive TCA pathway, which can be used to efficiently synthesize L‐malate (Figure 3) (Chi et al., 2016). However, the oxidative TCA pathway has six enzymes in the sub‐pathway from oxaloacetate to L‐malate, which release two molecules of CO_2_ in the process, limiting the carbon yield of L‐malate to 1 mol/mol glucose (Yin et al., 2015).

To obtain a higher yield of L‐malate, the reductive TCA (rTCA) cycle was developed based on pyruvate carboxylation and oxaloacetate reduction (Chi et al., 2016). Using this approach, the maximal yield of L‐malate can reached 1.44 g/g glucose based on fixing 1 mol of CO_2_ for 1 mol of L‐malate (Liu, Li, Du, et al., 2017; Liu, Li, Shin, et al., 2017; Liu, Xie, Shin, et al., 2017). For example, to engineer the industrial pyruvate production strain *Torulopsis glabrata* CCTCC M202019 to produce L‐malate, the key enzymes RoPYC, RoMDH, and SpMAE1 involved in the TCA cycle were overexpressed. The resulting strain achieved an L‐malate titre of 8.5 g/L, with a productivity of 0.14 g/L/h, which representing a remarkable 10‐fold increase compared to the control strain (Chen et al., 2013). Furthermore, modular pathway engineering, compartment engineering, and CO_2_ fixation were used to enhance the rTCA cycle. For modular pathway engineering, genes such as *pyc, mdh*, and *Spmae* were overexpressed to reinforce the biosynthesis and transport of L‐malate. Finally, the titre, yield, and productivity of L‐malate were increased to 30.75 g/L, 0.31 g/g, and 0.32 g/L/h in *S. cerevisiae* W4209, respectively (Chen et al., 2017). Compartment engineering can be used to avoid interference between enzymatic reactions. For example, the phosphoenolpyruvate carboxykinase and L‐malate dehydrogenase were targeted to the periplasmic space in a byproduct deleting strain to balance the cytoplasmic and periplasmic L‐malate biosynthesis pathways. Finally, the L‐malate titre yield and productivity respectively reached 25.86 g/L, 0.39 g/g and 0.36 g/L/h in a 5‐L fermenter (Guo et al., 2018). In another example, pyruvate carboxylase was targeted to the cytosol and mitochondria of *A. oryzae* to decrease the accumulation of the pathway intermediate pyruvate. As a result, the L‐malate titre reached 92.3 g/L in the cytoplasm and 90.5 g/L in mitochondria. After further regulating carbon and redox metabolism in the cytoplasm and mitochondria, the L‐malate titre reached 117.2 g/L, with a yield of 0.9 g/g corn starch and productivity of 1.17 g/L/h (Liu et al., 2018). CO_2_ fixation can be used to incorporate additional carbon from the co‐substrate to form more L‐malate. For example, the RuBisCO pathway was introduced along with the PCK pathway to fix CO_2_ for L‐malate synthesis in *E. coli*. Finally, the L‐malate titre, yield and productivity reached 51.86 g/L, 1.09 g/g and 0.72 g/L/h, respectively (Hu et al., 2018).

### Transporter engineering for improved L‐malate synthesis

Engineered transporters can efficiently improve L‐malate production, which was mainly implemented in *A. oryzae* and yeast (Figure 3) (Chen et al., 2017; Darbani et al., 2019). In *A. oryzae*, the AO090023000318 (C4T318) gene was screened among six potential transporters. When C4T318, *mdh3*, and *pyc* were overexpressed in *A. oryzae* NRRL 3488, the L‐malate titre, yield and productivity from glucose respectively reached 154 g/L, 1.03 and 0.94 g/L/h (Brown et al., 2013). In yeast, the C4‐dicarboxylate transporter *Spmae* gene and the related deubiquitination mechanism were regulated based on pathway enzymes to enhance the L‐malate titre, yield and productivity to 22.14 g/L, 0.22 g/g and 0.23 g/L/h, respectively. Furthermore, the expression of strength of the *mdh, pyc*, and *mae* genes was optimized using a modular strategy, which resulted in an L‐malate titre, yield and productivity of 30.25 g/L, 0.30 g/g and 0.32 g/L/h, respectively (Chen et al., 2017).

In addition, pathway compartmentalization can increase the concentration of key precursors of promote the carbon flux toward L‐malate, which was mainly implemented in mitochondria and carboxysomes. In *Candida glabrata*, the mitochondrial pyruvate carrier (MPC) can transport pyruvate into different subcellular compartments, and this active efflux can enhance the intracellular concentration of pyruvate, which is the direct precursor of L‐malate. Thus, the L‐malate titre could be improved by overexpressing this mitochondrial pyruvate carrier (Luo et al., 2018). In another interesting study, yeast mitochondrial membrane transporter Ctp1(p) was overexpressed to analyse the effect of the transporters on the production of L‐malate. In addition, the fungal Mae1 transporter from *A. carbonarius* was used to promote the secretion and transport of L‐malate. Finally, the overexpression of AcDct(p) increased the L‐malate titre 12‐fold (Darbani et al., 2019). In carboxysomes, the key CO_2_ fixation pathway can be sequestered to concentrate the CO_2_ and improve the carbon yield based on CO_2_ as a co‐substrate (Hammer & Avalos, 2017; Zhao et al., 2019). For example, the carboxysomes of the cyanobacterium *Prochlorococcus marinus* were reconstructed in *E. coli* to enhance the CO_2_ assimilation efficiency. Finally, ^13^C‐labelled metabolic flux analysis proved that this additional carbon flux can be used for the synthesis of central metabolites, such as L‐malate and fumarate (Zhang et al., 2021).

### Glyoxylate pathway for L‐malate synthesis

The glyoxylate shunt is a branch of the TCA cycle which directly determines the synthesis of L‐malate through this pathway. To improve the L‐malate synthesis efficiency, the glyoxylate pathway was modified by varying the gene expression levels to identify potential bottlenecks and then remove them. Furthermore, it was possible remove potential bottlenecks in the complete glyoxylate pathway (Deng et al., 2018). As a consequence, additional carbon flux was redirected into the glyoxylate pathway, making it available for fumarate and succinate production (Chen et al., 2020; Zhu et al., 2014).

Based on this, the glyoxylate cycle was utilized for L‐malate synthesis through modular pathway optimization combined with in vivo multiplexed CRISPRi tuning. In the glyoxylate cycle, the expression of enzymes such as PC, CS, CAN, ICL, and MS was regulated through CRISPRi to optimize the oxidative pathway for L‐malate synthesis. Finally, the engineered *E. coli* B0013‐47 achieve an L‐malate titre yield and productivity of 36 g/L, 0.63 g/g and 0.6 g/L/h, respectively (Gao et al., 2018). Furthermore, the carbon flux distribution between the TCA cycle and glyoxylate shunt can be balanced using advanced metabolic engineering tools.

### One‐step pathway for L‐malate synthesis

A one‐step pathway that uses malic enzyme to transform pyruvate into L‐malate can achieve a theoretical L‐malate yield of 2 mol/mol, which is identical to the yield of the reductive TCA cycle (2 mol/mol), and higher than that of the glyoxylate cycle (1.33 mol/mol) (Figure 3) (Dong et al., 2017; Zelle et al., 2008). Therefore, malic enzyme can convert pyruvate into L‐malate with no intermediates, which gives it great potential for the synthesis of L‐malate.

To implement the one‐step pathway, the cofactor preference of NADP(H)‐dependent malic enzyme had to be changed. Strains expressing the triple mutant R221G/K228R/I310V mutant of malic enzyme showed 1.2‐fold and 2.7‐fold improvements in L‐malate production, compared to that of strains using malic enzyme with NADPH and NADH as cofactors (Morimoto et al., 2014). Furthermore, the NADP^+^‐dependent malic enzyme mutant C490S was introduced in a byproduct deletion strain, and NADH kinase was overexpressed. Finally, the L‐malate titre, and productivity respectively reached 7.78 g/L and 0.11 g/L/h in shake flasks, as well as 21.65 g/L, and 0.3 g/L/h in a 5‐L fermenter, with a yield of 0.36 g/g (Dong et al., 2017). In a different approach, pyruvate kinase (PykF) and malic enzyme (SfcA) were assembled on the synthetic scaffold SH3D/L to enhance the close co‐localization of key pathway enzymes, which can increase the carbon flux from pyruvate to L‐malate in the one‐step pathway. The resulting strain reached an L‐malate titre and productivity of 30.2 g/L and 0.36 g/L/h in a 5‐L bioreactor, bioreactor (Somasundaram et al., 2018; Wei et al., 2021).

## FERMENTATION REGULATION FOR INCREASED L‐MALATE PRODUCTION

Fermentation regulation for increased L‐malate synthesis mainly include the process control, regulation of factors influencing the fermentation, and the addition of neutralizing agents, which can regulate the cellular metabolism to promote L‐malate production (Iyyappan, Baskar, et al., 2019; Iyyappan, Bharathiraja, et al., 2019; Ochsenreither et al., 2014). As in many other fermentation processes, the factors most directly affecting L‐malate production are dissolved oxygen, temperature and pH. The optimal temperature for L‐malate synthesis in bacteria, yeast and filamentous fungi is mainly between 25 and 37°C, depending on the characteristics of different species (Chi et al., 2016; Nakayama et al., 2012; Zou et al., 2019). In addition, the dissolved oxygen concentration can be influenced by changing the rotor speed and aeration rate. For example, when the agitation speed was optimized from 200 to 600 rpm, the L‐malate titre was increased to 83.3 g/L. Furthermore, the aeration rate was varied from 1 vvm to 3 vvm, and the L‐malate titre of *A. oryzae* FCD15 was increased to 105.5 g/L by applying the optimized aeration rate of 2 vvm (Chen et al., 2019).

In addition to these factors, the pH and sugar tolerance play an important role in L‐malate synthesis. For example, when crude glycerol and yeast extract were used as substrate, the initial pH value was regulated from 5, 6, 7 to analyse the actual factors affecting L‐malate synthesis in *A. niger*. Furthermore, the Student's *t*‐test and *F*‐test were performed using MINITAB to identify the optimal pH of 6, which resulted in an L‐malate titre of 91.4 g/L. An acidic environment may affect the pellet morphology and cell growth, which directly affects L‐malate synthesis (Iyyappan, Baskar, et al., 2019; Iyyappan, Bharathiraja, et al., 2019). When the pH was controlled by adding CaCO_3_, the L‐malate titre reached 132 g/L in shake flasks and 168 g/L in a 10‐L fermenter (Khan et al., 2014). Another interesting example is sugar tolerance. *Zygosaccharomyces rouxii* V19, which can growth in more than 10% glucose, was isolated and selected as a sugar‐tolerant yeast from high‐sugar fermented foods. Notably, an initial glucose concentration of 30% was used, which can repress contaminating bacteria. Under the optimal conditions (pH of 5 and temperature of 26°C), the L‐malate titre reached 74.9 g/L, with a yield of 0.55 g/g and productivity of 4.99 g/L/d (Taing & Taing, 2006).

The addition of neutralizing agents to the fermentation broth can remove the free acid and convert it into salts of L‐malate. The efficient production of L‐malate can be achieved by adding calcium carbonate, sodium hydroxide or other neutralizing agents during the fermentation process, because the pH of the fermentation broth can be controlled close to the optimum of the production strain. In addition, adding calcium carbonate can pull the flux of rTCA and increase the yield of L‐malate (Chi et al., 2016; Zambanini, Sarikaya, et al., 2016; Zambanini, Kleineberg, et al., 2016). However, the recovery of L‐malate results in large amounts of waste (gypsum) in the overall process. As a consequence, the direct production of the free acid is greatly preferred (less acid/base consumption and less salt waste), but gives lower titers, due to the weak tolerance of existing strains to the strong acid L‐malate (Liang et al., 2021; Liu, Li, Du, et al., 2017; Liu, Li, Shin, et al., 2017; Liu, Xie, Shin, et al., 2017). Therefore, it is often necessary to add a neutralizer to maintain pH balance during L‐malate fermentation.

## INDUSTRIAL APPLICATIONS FOR L‐MALATE

L‐malate is a natural dicarboxylic acid with important roles in both biology and the chemical industry (Li, Shen, et al., 2021; Li, Yang, et al., 2021; Sun et al., 2020). For applications in the food and pharmaceutical industries, L‐malate can be produced using engineered microorganisms, including *A. niger, A. oryzae, E. coli*, and *S. cerevisiae* (Li, Shen, et al., 2021; Li, Yang, et al., 2021; Liu, Li, Du, et al., 2017; Liu, Li, Shin, et al., 2017; Liu, Xie, Shin, et al., 2017). The potential market demand for L‐malate reached 200,000 tons in 2003, but the worldwide production capacity was only 40,000 tones (Chi et al., 2016; Kajiyama et al., 2003; Mondala, 2015). The market cap reached 176.32 million USD in 2015 (Iyyappan, Baskar, et al., 2019; Iyyappan, Bharathiraja, et al., 2019; Mondala, 2015). In addition to the food and pharmaceutical industry, L‐malate has applications as a colour fixative, as well as in metal cleaning and finishing (Mohan et al., 2012). In addition, L‐malate has applications in detoxification and beauty (Kim et al., 1997; Liu, Li, Du, et al., 2017; Liu, Li, Shin, et al., 2017; Liu, Xie, Shin, et al., 2017). Medically, L‐malate has an immune‐boosting effect and can be used as an antimicrobial agents(Kajiyama et al., 2003). In addition, it can be used for the treatment of hyperammonemia and liver disfunction (Liu, Li, Du, et al., 2017; Liu, Li, Shin, et al., 2017; Liu, Xie, Shin, et al., 2017).

Due to its acidity and a certain sweetness, malate can be used as an acidulant and flavour additive in beverages, chocolate, ice cream and other foods (Nakayama et al., 2012). In addition, malate can also be used for pH adjustment in yoghurt fermentation, as well as in conjunction with tartrate in wine brewing. L‐malate is widely used in wine, other beverages, jam, chewing gum and other foods due to its soft acidity and long shelf‐life. Because of its special organoleptic profile, it can effectively improve the flavour of fruit‐based food (Chi et al., 2016; Goldberg et al., 2006).

In addition to its applications in the food industry, L‐malate is a synthetic raw materials in the chemical industry, and it can also be used as a descaling agent and fluorescent whitening agent. L‐malate can be added to shellac varnishes or other varnishes to prevent the finish from crusting, while polyester resins and alkyds produced from this acid are plastics for special purposes (Sauer et al., 2008). L‐malate can also be used to produce PMA for biomedical use by direct polycondensation (Kajiyama et al., 2003). In addition, L‐malate is also used in the production of toothpaste and synthetic fragrances (Chi et al., 2016; Kim et al., 1997; Sauer et al., 2008).

In the pharmaceutical industry, L‐malate is added to various tablets and syrups to give them a fruity taste and facilitate absorption. Moreover, L‐malate can also be added to amino acid injections to treat hyperammonemia and liver dysfunction (Chi et al., 2016; Sauer et al., 2008). In addition, citrulline malate, sunitinib malate, and calcium citrate‐malate can be used to adjust the performances in resistance‐trained athletes, increasing the angiogenic and hypertrophic capacity, while reducing the risk of kidney stones (Battat et al., 2010; Glenn et al., 2015; Liu, Li, Du, et al., 2017; Liu, Li, Shin, et al., 2017; Liu, Xie, Shin, et al., 2017; Saber et al., 2016).

## CONCLUDING REMARKS AND FUTURE PROSPECTS

In this review, we comprehensively summarized the microbial biosynthesis of L‐malate from the aspects of chassis, substrate utilization, synthesis pathway, fermentation regulation and industrial applications. Although a large‐scale metabolic engineering strategy and microbial chassis strains were developed to efficiently synthesize L‐malate, there are still significant challenges facing the translation of microbial L‐malate production to the industrial scale. Therefore, future studies should further focus on the following aspects:

- Constructing a more efficient CO_2_ sequestration system. This can mainly be achieved by enhancing the CO_2_ fixation capacity and reducing CO_2_ emissions to further increase the carbon yield of L‐malate synthesis. This strategy can not only enhance the yield of the L‐malate pathway, but it can also relieve global warming. This approach can be implemented by increasing the ATP and NADH pool based on a light‐driven CO_2_ sequestration system to reinforce the L‐malate pathway yield. Finally, CO_2_ fixation combined with the primary substrate utilization achieved an L‐malate yield of 1.48 mol/mol (Hu et al., 2021).
- Developing natural and artificial organelles for enhancing the carbon flux in the L‐malate pathway. Natural organelles, such as the mitochondria, endoplasmic reticulum, Golgi and carboxysomes, can be engineered to concentrate enzymes and increase the metabolic flux of key pathways in chemical synthesis (Avalos et al., 2013; Hammer & Avalos, 2017; Huang et al., 2018). In addition, artifical membrane‐less organelle, artificial mitochondria and artificial chloroplasts can be designed to compartmentalize target enzymes and supplying a specific environment, which will be conducive to highly efficient enzymatic reactions (Li, Feng, et al., 2018; Wang et al., 2022; Xu, Fei, et al., 2019; Xu, Shan, et al., 2019). Based on the available studies, the L‐malate pathway can be further enhanced by designing natural and artificial organelles.
- Improving the tolerance of production strains to extracellular environmental stress. Osmotic stress and acid stress may be the key factors that limit L‐malate production, as they are intrinsic to L‐malate accumulation. Therefore, it is necessary to improve the stress resistance of chassis cells (Guan & Liu, 2020; Polizzi & Kontoravdi, 2015; Sun et al., 2018). In general, resistance to high osmotic pressure is a desirable phenotype for strains used in the production of organic acids (Kwon et al., 2011). L‐malate and succinate fermentations are both faced with high osmotic stress during the late stages of the fermentation process. Previous studies added sulfur‐containing amino acids or overexpressed copper efflux genes to improve osmotolerance. Finally, a *cusS* mutant was introduced to increase the cell mass and succinate titre by 120% and 492% (Xiao et al., 2017). In addition to acid stress, the membrane integrity, membrane fluidity and membrane permeability can be disturbed due to L‐malate accumulation, which affects the membrane composition and membrane functions (Qi et al., 2019). For example, the physiological functions and productive capacity of *C. glabrata* were confirmed to be affected by L‐malate accumulation, and a dynamic tolerance system was constructed based on the transcription factors CgUSV1 and CgYAP3, combined with the L‐malate‐driven promoter Pcgr‐10 to improve the strain robustness. The L‐malate titre and productivity of the final strain 012 respectively reached 35.5 g/L and 0.33 g/L/h by applying this dynamic tolerance system (Liang et al., 2021).
- Constructing intelligent genetic circuits for regulating L‐malate synthesis. The L‐malate pathway flux is affected due to byproduct synthesis and irrelevant carbon waste, which can affect cell growth and chemical production (Zelle et al., 2008). Thus, regulating carbon flux with intelligent genetic circuits could further improve L‐malate synthesis (Gupta et al., 2017; Hou et al., 2020; Soma & Hanai, 2015). Spatiotemporal switches can assist the pathway‐independent dynamic regulation of chemical production, mainly using visible light regulation, temperature regulation and oxygen regulation (Moser et al., 2018; Yu et al., 2022; Zhao et al., 2018). For example, the *pfkA* gene in the EMP pathway and *zwf* gene in the PPP pathway were repressed using thermosensors coupled with a CRISPRi system to regulate the carbon flux of cell growth and increase the 2′‐fucosyllactose titre to 28.2 g/L (Yu et al., 2022).

## AUTHOR CONTRIBUTIONS

Q. D. and C. Y. conceived and designed the manuscript. Q. D. provided and analysed literature. Q. D. wrote and revised the manuscript. Q. D. and C. Y. revised the manuscript. All authors read and approved the manuscript.

## CONFLICT OF INTEREST

The authors declare that they have no conflict of interest.

## ETHICAL APPROVAL

This article does not contain any studies with human participants or animals performed by any of the authors.
